# Tempeh-Derived Savory Powders as Ingredients to Compensate Sodium Reduction in Vegetable Broth

**DOI:** 10.3390/foods15132367

**Published:** 2026-07-03

**Authors:** Reggie Surya, Felicia Tedjakusuma, Aphinya Thinthasit, Frederico Richie Citra, Matthew Alexander Samiadji, David Nugroho

**Affiliations:** 1 Food Technology Department, Faculty of Engineering, Bina Nusantara University, Jakarta 11480, Indonesia; 2 Department of Food Science and Technology, Faculty of Agro-Industry, Kasetsart University, Bangkok 10900, Thailand; 3 Department of Integrated Science, Faculty of Science, Khon Kaen University, Khon Kaen 40002, Thailand

**Keywords:** tempeh, salt reduction, umami, sensory evaluation, consumer acceptance

## Abstract

Excessive sodium intake remains a major public health concern, yet reducing salt in savory foods often decreases consumer acceptance. This study evaluated tempeh-derived powders as natural flavor-enhancing ingredients for sodium reduction in vegetable broth. Four powder variants were prepared from laboratory-made tempeh: fresh tempeh powder (TP), overripe tempeh powder (OTP), roasted tempeh powder (RTP), and fried tempeh powder (FTP). These powders were incorporated into broth formulations with 20% and 30% sodium reduction, alongside full-salt and reduced-salt controls. Physicochemical analyses were conducted, and sensory evaluation was performed with 282 consumer panelists using consumer acceptance testing, perceived saltiness assessment, Rate-All-That-Apply (RATA), Just-About-Right (JAR), penalty analysis, and principal component analysis (PCA). Calculated added sodium decreased from 314.7 mg/100 mL in the full-salt control to 251.8 and 220.3 mg/100 mL in the 20% and 30% reduced-sodium controls, respectively. Tempeh powder addition significantly modified broth color and viscosity, while pH remained stable at 6.19–6.25. Total free amino-compound values increased markedly in tempeh-containing broths, with OTP showing the highest values at 245.6 and 242.2 mg glutamic acid equivalents/L in OTP_80 and OTP_70, respectively. Sensory results showed that OTP_80 achieved the highest overall hedonic score among reduced-sodium formulations and was significantly higher than CON_80 and CON_70 (*p* < 0.05). OTP_70 also maintained high overall liking and was not significantly different from the full-salt control. RATA and PCA indicated distinct sensory profiles among powder variants. These findings suggest that overripe tempeh powder may serve as a promising clean-label ingredient for sodium compensation in reduced-sodium savory broth.

## 1. Introduction

Excessive sodium intake remains a major public health concern because of its strong association with hypertension, cardiovascular disease, stroke, and chronic kidney disease. High sodium consumption is recognized as one of the leading dietary risk factors for premature mortality worldwide, and sodium reduction has been identified as a priority strategy for preventing non-communicable diseases (NCDs) [1,2,3]. Although salt is important for food palatability and preservation, average sodium intake in many populations remains well above recommended limits [4]. Therefore, reducing sodium in frequently consumed savory foods has become an important target for both health authorities and the food industry.

Broth is a particularly relevant model for sodium-reduction research because it is both a widely consumed food product and a versatile culinary ingredient [5]. Broth is commonly consumed directly as soups or clear savory beverages, but it is also widely used as a flavor base in sauces, gravies, noodles, rice dishes, stews, casseroles, ready meals, seasoning systems, and processed convenience foods. Commercial broth products, including bouillon cubes, stock powders, concentrated pastes, and liquid stocks, are widely used in households and foodservice settings because they provide convenient savory flavor and aroma. Due to this broad application, improving the nutritional quality of broth products may have impact beyond direct broth consumption, as reformulated broth ingredients can influence sodium intake across multiple food categories.

From a sensory perspective, broth is also a useful model because salt is one of its main drivers of palatability. In clear broth systems, salt contributes not only to saltiness, but also to overall flavor intensity, taste balance, and suppression of undesirable notes such as bitterness [6]. When sodium is reduced, broths often become flat, weak, or less satisfying, which may reduce consumer acceptance. This creates a major challenge for product reformulation, particularly when sodium reduction must be achieved without compromising sensory quality.

Several strategies have been explored to support sodium reduction in broth, including partial substitution with potassium salts, use of monosodium glutamate (MSG), yeast extracts, hydrolyzed vegetable proteins, herbs, spices, and aroma-based sensory enhancement [7,8,9,10]. However, some approaches may introduce metallic or bitter aftertastes, increase formulation complexity, or conflict with growing consumer demand for natural and clean-label ingredients. As a result, there is increasing interest in naturally fermented food ingredients that can provide savory taste and aroma while maintaining label simplicity.

Tempeh is a traditional Indonesian fermented soybean product produced primarily through fermentation by *Rhizopus* spp. During fermentation, soybean proteins are partially hydrolyzed, resulting in increased digestibility and formation of peptides and free amino acids that may contribute to umami and savory perception [11,12]. Tempeh is also increasingly recognized internationally as a sustainable plant-based protein source with functional potential [13].

Beyond fresh tempeh, different post-fermentation processing methods may substantially alter flavor characteristics. Fresh tempeh typically has mild nutty and beany notes. Overripe tempeh, locally known as *tempe semangit*, develops stronger fermented and savory aromas through extended fermentation and additional biochemical changes [14]. Thermal treatments such as roasting and frying may further enhance flavor through Maillard reactions, lipid oxidation, and thermal degradation pathways, generating roasted, nutty, and fried aroma notes associated with savory appeal [15,16]. These differences suggest that tempeh powders prepared using different processing methods may differ in their ability to compensate for sodium reduction in broth systems.

From a sensory perspective, sodium reduction should not be evaluated solely by measuring sodium content. It is equally important to determine whether consumers still perceive products as sufficiently salty, savory, and acceptable [6,17]. Consumer-based methods such as hedonic testing, Just-About-Right (JAR) scaling, penalty analysis, and Rate-All-That-Apply (RATA) profiling are particularly useful in reformulation studies. Hedonic testing measures overall liking, JAR scaling identifies whether saltiness is perceived as too low, ideal, or too high, while penalty analysis estimates the impact of non-optimal saltiness on liking. RATA profiling further provides rapid characterization of sensory attributes such as saltiness, umami, roasted aroma, fermented notes, bitterness, and aftertaste.

Although tempeh powder has been explored in soup formulations and tempeh-based seasoning powders [18,19], and umami-rich ingredients have been investigated as sodium-reduction tools [20,21], studies specifically evaluating different tempeh-derived powder variants as natural savory ingredients for sodium compensation in reduced-sodium broth remain limited. Therefore, the present study investigated the ability of four tempeh powder variants—fresh tempeh powder, overripe tempeh powder (*tempe semangit*), roasted tempeh powder, and fried tempeh powder—to support sodium reduction in a clear broth model. The broth was formulated at 100%, 80%, and 70% of the reference salt level, representing up to 30% sodium reduction. Consumer evaluation was conducted using hedonic testing, JAR scaling with penalty analysis, and RATA sensory profiling. It was hypothesized that selected tempeh powders, particularly roasted and overripe tempeh powders, would enhance savory perception and help maintain consumer acceptance under reduced-sodium conditions.

## 2. Materials and Methods

### 2.1. Materials

Yellow soybean seeds (*Glycine max* L. Merr.), cultivar Anjasmoro, were obtained from a local agricultural supplier in West Java, Indonesia. Soybeans originated from the same harvest lot to minimize variation in compositional variability that could influence fermentation behavior, texture development, and flavor generation during tempeh production. Commercial tempeh starter (brand “Raprima”, PT Aneka Fermentasi Industri, Bandung, Indonesia), containing *Rhizopus* spp., was used as the inoculum. Food-grade sodium chloride (NaCl) was purchased from a local retail supplier and used as the sole sodium source for broth formulation. Refined palm oil was used for the frying treatment during preparation of fried tempeh powder. Fresh vegetables used for broth preparation consisted of carrot, onion, celery, cabbage, and garlic, all purchased from a local supermarket in Jakarta, Indonesia on the day of broth preparation. Distilled water was used for all broth formulations and analytical procedures. For chemical analyses, analytical-grade reagents were used. Ninhydrin reagent, L-glutamic acid standard, Folin–Ciocalteu reagent, gallic acid standard, sodium carbonate, ethanol, and methanol were obtained from Sigma-Aldrich (St. Louis, MO, USA), Merck (Darmstadt, Germany), or equivalent analytical-grade suppliers. Buffer solutions at pH 4.00 and 7.00 for pH-meter calibration were obtained from Hanna Instruments (Woonsocket, RI, USA) or equivalent suppliers.

### 2.2. Production of Tempeh and Preparation of Tempeh Powder Variants

Tempeh was produced under controlled laboratory conditions as described previously [22]. Soybeans were washed thoroughly and soaked in potable water at a soybean-to-water ratio of 1:3 (*w*/*v*) for 16 h at room temperature (28 ± 2 °C). After soaking, the hydrated beans were manually dehulled by gentle rubbing and rinsing to remove the hulls. The dehulled soybeans were then boiled in water for 30 min, drained, and cooled to approximately 35 °C. Surface moisture was reduced by spreading the beans on stainless-steel trays for 20 min under clean air conditions, as excess moisture may encourage bacterial growth and interfere with proper mold development during fermentation. The cooled soybeans were then inoculated with tempeh starter at 0.15% (*w*/*w*) and mixed thoroughly to ensure uniform starter distribution. Approximately 250 g of inoculated soybeans were packed into perforated polyethylene bags with 1 mm perforation spacing. The bags were incubated at 30 ± 1 °C for 48 h until compact white mycelial growth was observed and the beans were fully bound together. This product was designated as fresh tempeh. Three independent fermentation batches were prepared on separate days and treated as biological replicates.

Fresh tempeh from each fermentation batch was divided into four portions to prepare fresh tempeh powder (TP), overripe tempeh powder (OTP), roasted tempeh powder (RTP), and fried tempeh powder (FTP). For TP, fresh tempeh was cut into cubes of 1 cm × 1 cm × 1 cm, dried in a hot-air oven at 55 °C for 12 h until moisture content was below 10%, cooled to room temperature, milled, and sieved through a 60-mesh sieve. For OTP, fresh tempeh was further incubated at 30 ± 1 °C for an additional 48 h to obtain overripe tempeh (*tempe semangit*). No visible signs of contamination were observed in the samples. The overripe tempeh was then dried at 55 °C for 12 h, milled, and sieved. For RTP, fresh tempeh cubes were roasted in a preheated hot-air oven at 160 °C for 25 min, with manual turning after 12 min to ensure even heating and browning. The roasted samples were cooled and further dried at 55 °C for 6 h before milling and sieving. For FTP, fresh tempeh cubes were deep-fried in refined palm oil at 170 °C for 4 min until golden brown. Excess oil was removed using absorbent paper, after which the fried samples were dried at 55 °C for 6 h, milled, and sieved. All powders were packed in laminated aluminum pouches, sealed, and stored at 4 °C until use. The powders were used within two weeks of preparation.

### 2.3. Broth Formulation and Sodium Reduction Design

A clear vegetable broth model was selected as the food system because broth is widely consumed directly and also serves as a versatile base ingredient in soups, noodles, sauces, gravies, rice dishes, stews, ready meals, and seasoning applications. In addition, broth provides a relatively simple savory matrix in which changes in saltiness, umami, and overall flavor perception can be more clearly detected than in complex multi-component foods [5,6].

Based on an established recipe [23], the vegetable broth base was prepared fresh on each day of analysis using carrot (40 g/L), onion (40 g/L), cabbage (30 g/L), celery (15 g/L), shallot (5 g/L), and garlic (5 g/L). The vegetables were washed, cut into small and pieces of uniform size, combined with distilled water, and simmered in boiling water at 100 °C for 30 min. The broth was then filtered through a stainless-steel sieve followed by muslin cloth to obtain a clear liquid. The filtrate was cooled to approximately 60 °C before sodium adjustment and sample preparation. To minimize variability in the broth base, vegetables were purchased from the same supplier on each preparation day, weighed according to fixed ingredient-to-water ratios, and cut into pieces of uniform size before cooking. The same cooking temperature, simmering time, vessel type, and filtration procedure were applied for all batches.

Sodium chloride was added separately to the broth base to ensure that sodium level was the only variable altered across formulations, while the background flavor matrix remained constant. The reference formulation (100% salt level) contained 0.80% (*w*/*v*) NaCl, selected based on preliminary trials as a consumer-acceptable salt concentration for savory broth systems. Two reduced-sodium formulations were subsequently prepared: 0.64% NaCl (80% salt level; 20% sodium reduction) and 0.56% NaCl (70% salt level; 30% sodium reduction).

The concentration of tempeh powder was selected based on preliminary formulation trials using 1.0%, 2.0%, and 3.0% (*w*/*v*) powder addition. At 1.0%, the sensory contribution of tempeh powder was relatively weak and insufficient to clearly differentiate the treated broths from the reduced-sodium controls. At 3.0%, the broths became noticeably more turbid and viscous, with stronger tempeh-derived notes and greater sedimentation during serving. Therefore, 2.0% (*w*/*v*) was selected as a balanced concentration that provided detectable savory sensory contribution while maintaining acceptable broth appearance, fluidity, and suitability for consumer evaluation.

Fresh tempeh powder (TP), overripe tempeh powder (OTP), roasted tempeh powder (RTP), and fried tempeh powder (FTP) were each tested at both reduced-sodium levels. Control broths without tempeh powder were prepared at 100%, 80%, and 70% salt levels. Accordingly, eleven formulations were prepared in this study: CON100, CON80, CON70, TP80, TP70, OTP80, OTP70, RTP80, RTP70, FTP80, and FTP70. The composition of all broth formulations used in this study, including sodium chloride levels and tempeh powder treatments, is presented in Table 1. For each formulation, the required amount of NaCl was first dissolved in the warm broth base, followed by gradual addition of tempeh powder under magnetic stirring for 5 min to ensure homogeneous dispersion. All samples were reheated to 85 °C for 5 min to allow flavor release and hydration, then filtered through stainless-steel mesh to remove coarse particles. Samples were held in insulated containers and served warm at approximately 60 °C during sensory evaluation.

All broth samples used within the same testing session were freshly prepared on the day of evaluation using identical preparation procedures. Preliminary observations confirmed that the selected tempeh powder concentration did not cause excessive sedimentation or unacceptable visual instability during the serving period.

### 2.4. Physicochemical Analysis

#### 2.4.1. Color Measurement

The color of each broth sample was determined using a chromameter (CR-400, Konica Minolta, Singapore) and expressed in the CIE L*a*b* color space. The L* value indicates lightness from black to white, while a* represents the red–green axis and b* represents the yellow–blue axis. Measurements were performed in triplicate using a 10 mm optical path-length glass cuvette. The color difference (ΔE*) represents the Euclidean distance between two color points in the CIE L*a*b* color space [24] and was calculated for each replicate measurement relative to the mean L*, a*, and b* values of the full-salt control broth (CON_100), using the following equation: ΔE* = [(ΔL*)^2^ + (Δa*)^2^ + (Δb*)^2^]^0.5^. The ΔE* values were then expressed as mean ± SD.

#### 2.4.2. pH Analysis

The pH of the broth samples was measured at room temperature (25 °C) using a digital pH-meter (HI98107, Hanna Instruments, Woonsocket, RI, USA). The instrument was calibrated daily with standard buffer solutions at pH 4.00 and 7.00. Measurements were carried out in triplicate and expressed as mean ± standard deviation.

#### 2.4.3. Apparent Viscosity

Apparent viscosity was measured using a rotational viscometer (DV2T, Brookfield AMETEK, Middleboro, MA, USA) at room temperature (25 °C) and 60 rpm. Approximately 100 mL of each broth sample was placed in the measurement chamber and allowed to equilibrate for 2 min before measurement. Results were expressed as mPa·s. Each formulation was analyzed in triplicate.

#### 2.4.4. Calculation of Added Na and Determination of Total Free Amino Compounds

Calculated added sodium (Na) was estimated from the amount of sodium chloride (NaCl) added to each broth formulation. The sodium contribution from NaCl was calculated using the molecular weight ratio of sodium to sodium chloride, assuming that NaCl contains 39.34% sodium by mass. Therefore, the calculated sodium contents of the 0.80%, 0.64%, and 0.56% NaCl formulations were 314.7, 251.8, and 220.3 mg sodium/100 mL, respectively. Sodium content was not determined by instrumental mineral analysis.

Total free amino acids were determined using the ninhydrin colorimetric method with slight modification [25]. Briefly, 1 mL of broth sample was centrifuged at 5000× *g* for 10 min to remove suspended particles. Then, 1 mL of the clear supernatant was mixed with 1 mL of ninhydrin reagent and heated at 100 °C for 15 min. After cooling to room temperature, 5.0 mL of ethanol–water diluent (50:50, *v*/*v*) was added. The absorbance was measured at 570 nm using a UV–Vis spectrophotometer (UV5, Mettler-Toledo, Greifensee, Switzerland). L-glutamic acid was used as the standard, and the results were expressed as mg glutamic acid equivalents per liter (mg GAE/L).

### 2.5. Sensory Evaluation

#### 2.5.1. Consumer Panel Recruitment and Testing Conditions

Sensory evaluation was conducted using untrained consumer panelists recruited from the university community and surrounding area. Participants were eligible if they were at least 18 years old, regularly consumed savory broth, soup, or noodle-based products, and had no self-reported taste or smell disorders. Individuals with known soybean allergy or dietary restrictions preventing sample consumption were excluded from participation. All panelists provided informed consent prior to the test. The use of consumer panelists was considered appropriate since the objective of the study was to evaluate product acceptability and sensory perception of reduced-sodium broth formulations under realistic consumption conditions. The study protocol has been reviewed and approved by the Ethical Research Committee of Research and Technology Transfer Office (RTTO) at Bina Nusantara University (067/VRRTT/IV/2026, 1 April 2026).

A total of 282 untrained consumers participated in the sensory evaluation. The panel consisted of 128 male (45.4%) and 154 female participants (54.6%). Most participants were young adults, with 51.8% aged 18–24 years and 27.7% aged 25–34 years, while smaller proportions were aged 35–44 years (12.1%) or ≥45 years (8.4%). Product familiarity was recorded to support the relevance of the sensory evaluation. Most panelists regularly consumed broth or soup, with 46.8% reporting consumption at least three times per week and 36.9% reporting consumption one to two times per week. Tempeh was also familiar to the participants, with 34.0% consuming it at least three times per week and 41.8% consuming it one to two times per week.

Sensory sessions were conducted in individual booths under controlled ambient lighting and temperature. Broth samples were freshly prepared on the day of testing and maintained at approximately 50 °C prior to serving. Sensory evaluation was conducted under blind conditions. Panelists were informed that they would evaluate warm vegetable broth samples, but they were not informed about the salt level, sodium-reduction status, or the presence or type of tempeh powder in each formulation. Each panelist evaluated all 11 broth formulations one-by-one in a single sensory session. To reduce fatigue, adaptation, and carry-over effects, samples were served in small portions of approximately 30 mL following a randomized and balanced presentation order. Panelists were instructed to rinse their mouth with room-temperature drinking water between samples and to take a short break after every three to four samples or whenever needed before continuing the evaluation.

#### 2.5.2. Hedonic Evaluation

Overall liking was evaluated using a 9-point hedonic scale, ranging from 1 = “dislike extremely” to 9 = “like extremely” [26]. Panelists were asked to rate each broth sample based on their overall sensory impression, including taste, aroma, mouthfeel, and aftertaste. This evaluation was used to determine whether the addition of tempeh powders could maintain consumer acceptance when the sodium level was reduced.

#### 2.5.3. Just-About-Right (JAR) Saltiness Evaluation and Penalty Analysis

Perceived saltiness adequacy was assessed using a 5-point Just-About-Right (JAR) scale, consisting of “much too low,” “slightly too low,” “just-about-right,” “slightly too high,” and “much too high” [27]. For statistical analysis, responses were grouped into three categories: “too low” (scores 1–2), “JAR” (score 3), and “too high” (scores 4–5). The JAR scale was included to determine whether sodium reduction altered saltiness perception away from the consumer’s ideal level.

Penalty analysis was performed by combining JAR saltiness responses with overall liking scores. For each sample, the mean liking score of panelists who rated saltiness as just-about-right was compared with the mean liking score of panelists who rated saltiness as either too low or too high. The difference between these values was defined as the mean drop in liking. Weighted mean drops were subsequently calculated by considering both the magnitude of the liking decrease and the proportion of consumers selecting each non-JAR category. This method was used to determine whether non-optimal saltiness caused a meaningful reduction in liking and to identify which tempeh powder treatments were most effective in minimizing the sensory penalty associated with sodium reduction [28].

#### 2.5.4. Rate-All-That-Apply (RATA) Sensory Profiling

Consumer sensory profiling was carried out using the Rate-All-That-Apply (RATA) method [29]. In this approach, panelists were provided with a predefined list of sensory attributes and asked to rate the intensity of every attribute perceived in each sample using a 0–10 scale, where 0 indicated “not perceived” and 10 indicated “extremely intense.” Attributes not selected by the panelist were recorded as zero. The attribute list consisted of salty, umami/savory, sweet, sour, bitter, roasted aroma, fermented aroma, nutty aroma, beany flavor, grassy flavor, meat-like flavor, oily mouthfeel, and rancid note. These attributes were selected to capture the main taste characteristics of the broth, tempeh-related flavor notes, aroma changes associated with roasting and frying, and possible off-notes resulting from extended fermentation or lipid oxidation.

### 2.6. Statistical Analysis

All physicochemical and chemical analyses were performed in triplicate, and the results were expressed as mean ± standard deviation. Data normality and homogeneity of variance were checked prior to statistical analysis. Differences among broth formulations were evaluated using one-way analysis of variance (ANOVA), followed by Tukey’s honestly significant difference (HSD) post hoc test when significant differences were detected. Statistical significance was set at *p* < 0.05.

Sensory data obtained from 282 consumer panelists were analyzed separately for hedonic liking, JAR saltiness, and RATA sensory attributes. Overall liking scores were expressed as mean ± standard error of the mean (SEM). Differences in overall liking among samples were analyzed using one-way ANOVA followed by Tukey’s HSD test. For RATA data, non-selected attributes were coded as zero, and attribute intensity scores were treated as continuous data. Mean RATA intensity values were compared among samples using one-way ANOVA followed by Tukey’s HSD test.

JAR responses for saltiness were grouped into three categories: “too low,” “just-about-right,” and “too high.” The distribution of panelists in each category was expressed as percentage. Penalty analysis was conducted by comparing the mean overall liking score of panelists who rated saltiness as just-about-right with those who rated it as too low or too high. The mean drop in liking was calculated for each non-JAR category, and weighted mean drops were obtained by multiplying the mean drop by the percentage of panelists in the corresponding category.

To further explore relationships among sensory variables, an exploratory correlation analysis was performed using formulation-level mean values (n = 11 formulations). Pearson correlation coefficients (r) were calculated among overall liking, perceived saltiness intensity, JAR saltiness proportion, weighted mean drop from penalty analysis, and selected RATA attributes. The coefficient of determination (R^2^) was calculated as r^2^ to estimate the proportion of variation shared between paired variables. This analysis was used to support interpretation of sensory trends and was not intended to establish causal relationships.

Principal component analysis (PCA) was performed using the mean RATA intensity scores to visualize the relationship between broth formulations and sensory attributes. Prior to PCA, RATA data were mean-centered and standardized to give equal weight to all attributes. All statistical analyses were conducted using GraphPad Prism version 10.0 (GraphPad Software, San Diego, CA, USA) and XLSTAT (Addinsoft, Paris, France).

## 3. Results

### 3.1. Tempeh Powders Modified the Physicochemical Characteristics of Vegetable Broth

The physicochemical properties of the reduced-sodium broth samples supplemented with different tempeh powders are presented in Table 2 and Table 3. Distinct differences were observed in appearance, viscosity, and flavor-related chemical composition, whereas pH remained stable across treatments.

Lightness (L*) values were highest in the control samples (62.4–62.8), indicating a brighter and clearer broth appearance. The addition of tempeh powders significantly reduced L* values (*p* < 0.05), producing darker broths. Among the treatments, roasted tempeh powder generated the darkest samples (RTP_80 and RTP_70), followed by fried and overripe tempeh powders. Fresh tempeh powder caused a milder reduction in lightness. This trend was accompanied by increases in redness (a*) and yellowness (b*), particularly in the roasted samples, which also showed the highest a* and b* values. These findings suggest that thermal processing promoted brown-yellow pigments and color development that were subsequently transferred into the broth system.

The total color difference (ΔE*) further confirmed these visual changes. RTP samples showed the largest ΔE* values relative to CON_100, followed by FTP, OTP, and TP. This indicates that roasting had the strongest effect on visual appearance, whereas fermentation alone produced more moderate color modification.

Viscosity values ranged from 2.02 to 2.68 mPa·s. The controls had the lowest viscosity, while all tempeh-containing formulations were significantly thicker (*p* < 0.05). The greatest viscosity was observed in the fried tempeh treatments, followed by overripe and roasted tempeh powders. Fresh tempeh powder caused a smaller but still measurable increase. The increase in apparent viscosity may also have sensory relevance. Although the absolute viscosity values remained low and the samples were still within the range of fluid broth systems, tempeh powder addition significantly increased viscosity compared with the control broths. This effect may be attributed to the presence of dispersed solids, proteins, dietary fiber, and, particularly in the fried treatment, lipid-associated components from the tempeh powders. A slightly higher viscosity could contribute to greater perceived body, mouth-coating, and flavor persistence, which may partly explain why tempeh-containing broths were generally more acceptable than reduced-sodium controls.

No significant differences in pH were detected among samples, with values ranging from 6.19 to 6.25. This indicates that the incorporation of tempeh powder at the applied level did not substantially alter the acid–base balance of the broth matrix. Therefore, the main physicochemical changes associated with tempeh powder addition were more evident in color, viscosity, sodium level, and amino-compound-related parameters than in pH.

As designed, sodium content decreased according to salt reduction level. CON_100 contained 314.5 mg Na/100 mL, while CON_80 and CON_70 were reduced to 251.6 and 220.2 mg Na/100 mL, respectively. Tempeh powder addition did not materially change sodium concentration within the same salt level, confirming that sodium reduction was controlled by NaCl adjustment rather than by powder supplementation.

Marked differences were found in total free amino compounds, expressed as glutamic acid equivalent. Control broths contained the lowest concentrations, whereas all tempeh powder treatments significantly increased both parameters (*p* < 0.05). Overripe tempeh powder produced the highest values, followed by roasted, fried, and fresh tempeh powders. The OTP samples contained approximately threefold higher free amino acids than the controls, together with the highest glutamic acid concentration. These results suggest that extended fermentation enhanced proteolysis and released flavor-active compounds associated with umami perception. Roasting and frying also increased these compounds, although to a lesser extent than overripe fermentation.

Overall, the physicochemical data indicate that tempeh powders modified broth appearance and mouthfeel while simultaneously enriching the system with amino acid-based taste compounds that may contribute to sodium reduction strategies.

### 3.2. Overripe Tempeh Powder Showed the Most Consistent Improvement in Perceived Saltiness and Consumer Liking Under Sodium Reduction

**Figure 1:**
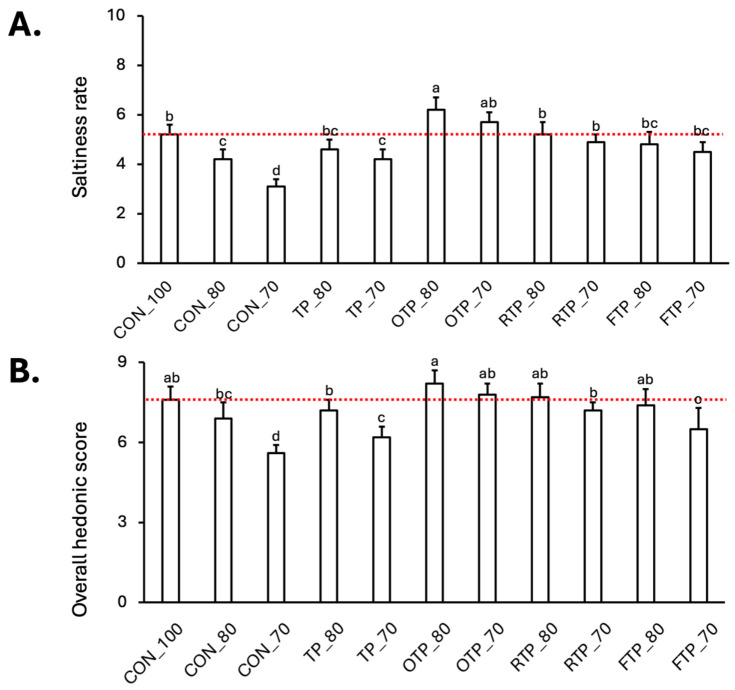
Perceived saltiness and consumer acceptance of reduced-sodium vegetable broth samples with tempeh powder variants. (**A**) Perceived saltiness intensity of broth samples evaluated using a 0–10 intensity scale. (**B**) Overall liking scores of broth samples evaluated using a 9-point hedonic scale. Values are presented as mean ± SEM (n = 282). Different letters above bars indicate significant differences among samples (*p* < 0.05). The red dashed line represents the value of CON_100, shown as a reference for comparison across formulations. Abbreviations: CON = control; TP = fresh tempeh powder; OTP = overripe tempeh powder; RTP = roasted tempeh powder; FTP = fried tempeh powder.

Perceived saltiness intensity and overall liking are presented in Figure 1. As expected, reducing NaCl concentration in the control broth decreased perceived saltiness, with CON_100 receiving the highest saltiness score and CON_70 receiving the lowest score among the controls. This confirms that consumers were sensitive to the sodium reduction levels applied in the present formulation design.

A similar trend was observed for overall liking. CON_100 received high acceptance, whereas CON_70 showed the lowest liking among the control samples. OTP_80 produced the highest liking score among the reduced-sodium formulations and was comparable to, or higher than, the full-salt reference. OTP_70 also maintained favorable acceptance despite the 30% sodium reduction. RTP and FTP samples showed intermediate liking values, while TP samples improved liking relative to reduced-sodium controls but less strongly than the processed tempeh variants. These results suggest that consumer acceptance was influenced not only by saltiness intensity, but also by flavor complexity and savory enhancement provided by the tempeh powders.

### 3.3. Different Tempeh Powder Variants Created Distinct Sensory Signatures of Vegetable Broth

The sensory consequences of using different tempeh powders were further explored through the RATA and principal component analysis. These complementary approaches provided deeper insight into how each treatment modified aroma, flavor, and mouthfeel characteristics of the reduced-sodium broth system. While hedonic and JAR analyses identified the most preferred formulations, the multivariate sensory results helped explain the sensory mechanisms behind those preferences.

**Figure 2:**
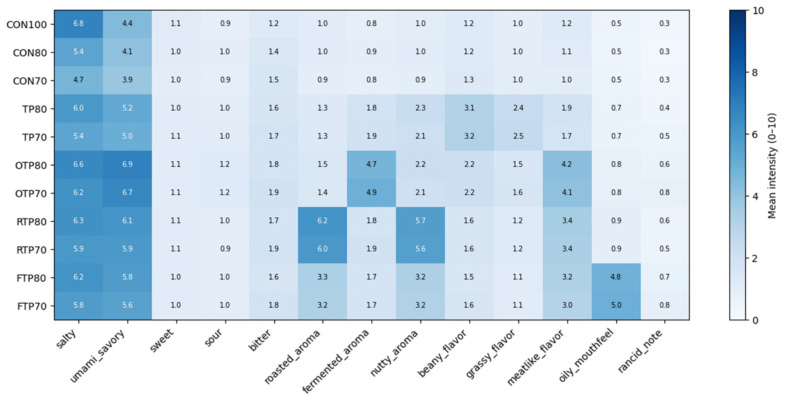
Heatmap of RATA sensory attributes of reduced-sodium vegetable broth samples with tempeh powder variants.

Figure 2 demonstrates that each powder treatment generated a distinct sensory fingerprint. The control broths showed the simplest profile, being dominated mainly by salty perception with relatively low intensities for most aroma and flavor descriptors. As sodium concentration decreased from CON_100 to CON_80 and CON_70, the salty attribute declined progressively. Lower-salt controls also tended to show reduced umami/savory intensity, suggesting that sodium reduction diminished not only saltiness but also overall flavor fullness. This simplified profile is consistent with the lower liking scores observed for the reduced-sodium controls.

Fresh tempeh powder (TP) shifted the profile toward plant-derived descriptors. TP samples showed higher beany flavor and grassy flavor intensities than the controls, together with moderate increases in umami/savory character. These attributes likely reflect the intrinsic soybean notes of standard tempeh that has undergone normal fermentation without additional processing. The increase in savory perception indicates that fresh tempeh still contributed taste-active compounds, although the persistence of beany and grassy notes may have limited its overall consumer appeal compared with the other processed variants.

Overripe tempeh powder (OTP) produced one of the most intense and complex sensory profiles. OTP samples were characterized by the highest fermented aroma, umami/savory, and meat-like flavor scores among all treatments. This result is highly consistent with the chemical composition data (Table 3), where OTP contained the greatest concentrations of total free amino compounds. Importantly, the elevated fermented aroma did not appear detrimental at the tested concentration, as OTP remained among the most accepted formulations. Instead, these matured notes may have functioned as desirable savory cues that increased flavor richness and compensated for sodium reduction.

Roasted tempeh powder (RTP) was mainly characterized by roasted and nutty aroma attributes, while fried tempeh powder (FTP) was more strongly associated with oily mouthfeel and richer sensory perception. These differences indicate that post-fermentation processing affected the sensory profile of tempeh powder in the broth system. However, because volatile compounds were not analyzed, the chemical basis of these aroma differences should be interpreted cautiously.

Across all treatments, rancid note intensity remained low, including in the fried samples. This is an important quality indicator, suggesting that neither the frying treatment nor subsequent storage caused strong oxidative off-flavors under the present conditions. Likewise, bitterness and sourness remained relatively low to moderate across formulations, indicating that sodium reduction and tempeh powder incorporation did not introduce severe undesirable tastes.

Overall, the RATA results indicate that tempeh powders may support sodium reduction through different sensory profiles. OTP was associated mainly with fermented, umami, and meat-like descriptors; RTP with roasted and nutty descriptors; FTP with oily mouthfeel; and TP with milder beany and grassy notes. These descriptive sensory differences provide a basis for the broader interpretation of sodium-reduction performance discussed in the following section.

**Figure 3:**
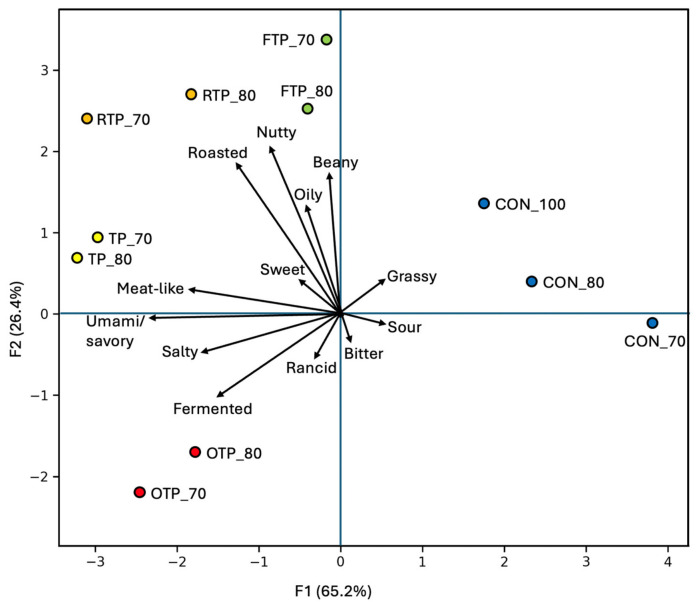
Principal component analysis biplot of RATA sensory attributes.

The PCA biplot (Figure 3) summarized the relationships among broth formulations and RATA sensory attributes. The first two principal components explained 91.6% of the total variance, with PC1 and PC2 accounting for 65.2% and 26.4%, respectively. Control samples were located on the positive side of PC1 and were separated from most tempeh-containing formulations, indicating that tempeh powder addition substantially altered the sensory profile of the broth matrix.

The tempeh-containing samples were distributed mainly on the negative side of PC1, but their positions differed according to powder type. TP samples were positioned closer to the umami/savory, meat-like, and salty vectors, suggesting that fresh tempeh powder contributed mild savory taste characteristics in the broth system. OTP samples were located in the lower-left region of the biplot and were more closely associated with fermented aroma and savory-related attributes, consistent with their stronger fermented sensory character. RTP samples were positioned toward the roasted and nutty vectors, indicating a sensory profile dominated by roasted/nutty attributes. FTP samples appeared in the upper region of the biplot and were more closely associated with beany, nutty, and oily mouthfeel attributes. Overall, the PCA results support the RATA heatmap by showing that different tempeh powder variants produced distinct consumer-perceived sensory profiles in reduced-sodium broth.

### 3.4. Overripe Tempeh Powder Minimized the Penalty Associated with “Too Low” Saltiness

**Figure 4:**
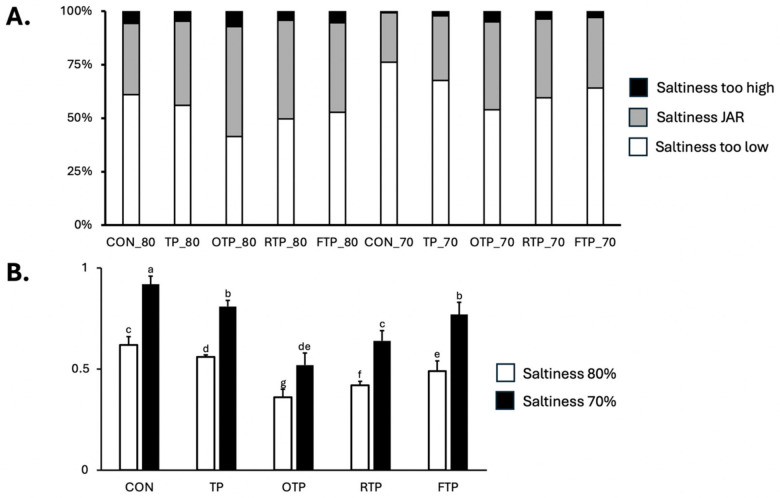
Saltiness adequacy and penalty analysis of reduced-sodium vegetable broth samples. (**A**) Distribution of saltiness Just-About-Right (JAR) responses, expressed as the percentage of panelists rating saltiness as too low, just-about-right, or too high. (**B**) Penalty analysis showing weighted mean drops in overall liking associated with non-JAR saltiness responses. Different letters above bars indicate significant differences in total weighted mean drop among samples based on one-way ANOVA followed by Tukey’s HSD post hoc test (*p* < 0.05). Abbreviations: CON = control; TP = fresh tempeh powder; OTP = overripe tempeh powder; RTP = roasted tempeh powder; FTP = fried tempeh powder.

Saltiness adequacy was further evaluated using JAR analysis, while the effect of non-ideal saltiness on consumer acceptance was assessed through penalty analysis (Figure 4). As expected, the full-salt control (CON_100) generated the highest proportion of “just-about-right” responses, confirming that the reference broth was perceived as appropriately seasoned. In contrast, reducing sodium without flavor enhancement shifted responses toward the “too low” category, particularly in CON_70, indicating that consumers clearly detected insufficient saltiness at the highest reduction level.

The addition of tempeh powders improved saltiness adequacy in all reduced-sodium formulations. Compared with the control broths at the same salt level, tempeh-containing samples generally showed lower percentages of “too low” responses and higher JAR proportions. OTP treatments consistently performed well, particularly OTP_80 and OTP_70, suggesting that compounds generated during extended fermentation supported perceived saltiness and flavor adequacy. RTP and FTP also showed favorable JAR distributions, indicating that roasted aroma cues and richer mouthfeel may contribute to improved saltiness adequacy. TP provided a smaller but still measurable benefit relative to the controls.

Penalty analysis supported these findings by showing that reduced-sodium controls experienced the greatest liking losses when consumers perceived saltiness as too low. CON_70 exhibited the highest weighted mean drop, confirming that insufficient saltiness substantially reduced acceptance. In contrast, tempeh powder supplementation lowered the penalty values across treatments, demonstrating its protective effect against liking loss under sodium reduction.

Among the flavor-enhanced samples, OTP generally ranked among the most effective treatments, with low penalty values at both salt levels. RTP and FTP also performed competitively in several cases, suggesting that aroma-driven enhancement and mouthfeel effects can partially compensate for lower sodium. TP reduced penalties more modestly, indicating weaker but still useful functionality. These results imply that different tempeh processing methods may restore saltiness through distinct sensory mechanisms rather than through a single pathway.

Overall, the combined JAR and penalty analyses confirm that tempeh powders can substantially mitigate the sensory disadvantages of sodium reduction in broth systems. Overripe tempeh powder showed the most consistent performance, while roasted and fried tempeh powders also provided strong alternative strategies for maintaining perceived saltiness and consumer acceptance.

## 4. Discussion

The present study shows that tempeh-derived powders can be used as practical flavor-building ingredients to support sodium reduction in savory broth systems. Rather than functioning simply as protein-rich additions, the powders contributed different combinations of taste, aroma, and mouthfeel attributes that helped restore the sensory quality of reduced-sodium formulations. This is important from a product development perspective because lowering salt often reduces perceived flavor intensity and consumer liking. The findings therefore suggest that sodium reduction may be more successful when it is approached as a broader flavor-design challenge, not merely as the removal or replacement of NaCl.

The health relevance of this strategy is clear. The World Health Organization recommends that adults consume less than 2 g of sodium per day, equivalent to approximately 5 g of salt per day [30]. However, sodium intake in many populations remains above this recommendation, largely because of processed foods, instant meals, sauces, seasonings, snacks, and foods consumed outside the home [4]. Excess sodium intake is strongly linked with elevated blood pressure and increased risk of cardiovascular disease, stroke, and kidney disease [1,2,3]. Therefore, reducing sodium in frequently consumed commercial products remains an important public health priority.

In this context, the present results support the possibility of achieving meaningful salt reduction in commercial savory products while maintaining sensory acceptance. In the broth model, tempeh powder addition improved perceived saltiness, overall liking, JAR saltiness responses, and penalty outcomes under reduced-sodium conditions. This is particularly relevant because small reductions across multiple product categories can accumulate into substantial reductions in daily sodium intake. A 20–30% reduction in salt may seem modest at the product level, but if applied across commonly consumed foods such as instant noodles, soup bases, sauces, and seasoning powders, the public health impact could be considerable.

Among the tested powders, overripe tempeh powder showed the most consistent performance. Its effectiveness was likely related to its higher free amino acid and glutamic acid contents, together with its fermented savory character. These components may enhance umami perception, increase flavor fullness, and make the broth taste more complete despite lower sodium content [31]. This suggests that controlled overripe tempeh, or *tempe semangit*, may have value beyond traditional culinary use. When standardized and applied at appropriate levels, it may function as a natural savory enhancer for reduced-sodium products.

Overripe tempeh powder (OTP) likely benefited from continued biochemical reactions during extended fermentation. After standard tempeh maturation, fungal enzymes such as proteases, peptidases, lipases, and carbohydrases may remain active, continuing to hydrolyze soybean macromolecules into smaller taste-active compounds [32]. Protein breakdown increases free amino acids and short peptides, while lipid hydrolysis may release free fatty acids that can subsequently participate in aroma formation. These reactions often intensify savory, matured, and fermented notes. In the present study, OTP showed the highest total free amino acid and glutamic acid contents, which supports this mechanism. Glutamic acid is particularly important because it is one of the key contributors to umami taste and can enhance the perception of fullness and seasoning in savory foods [33].

Extended fermentation may also generate more complex volatile compounds. Previous studies on fermented soybean foods have shown that continued microbial metabolism can increase aldehydes, alcohols, ketones, esters, sulfur-containing compounds, and low-molecular-weight acids, many of which contribute to matured savory aroma, pungency, or depth depending on concentration [34]. In Indonesian culinary practice, overripe tempeh (*tempe semangit*) is traditionally used in traditional dishes specifically because of its stronger flavor [35]. Therefore, the positive performance of OTP in this study is consistent with traditional empirical knowledge: controlled over-ripening transforms tempeh from a protein food into a seasoning ingredient.

The improved performance of OTP-containing broths should be interpreted as a sodium compensation effect rather than direct evidence of a confirmed sodium compensation effect. Although OTP_80 and OTP_70 contained less added sodium than the full-salt control, these formulations received higher perceived saltiness adequacy and consumer liking than the corresponding reduced-sodium controls. This effect may have resulted from the combined contribution of umami/savory perception, fermented sensory cues, flavor complexity, and improved balance in the broth matrix. Therefore, OTP did not necessarily restore sodium-derived saltiness itself but rather helped compensate for the sensory losses associated with sodium reduction. A product rich in free amino acids, peptides, and fermented savory volatiles may compensate by increasing total sensory stimulation [36]. This could explain why OTP improved both perceived saltiness and overall liking despite containing less added salt. Similar effects have been reported for naturally fermented ingredients such as soy sauce, fish sauce, yeast extract, and miso, which are frequently used to enhance low-sodium foods through combined taste and aroma pathways [37].

Roasted and fried tempeh powders also showed promising functionality, although through somewhat different sensory pathways. Roasted tempeh powder contributed roasted and nutty notes, which may increase the perception of warmth, depth, and cooked savory flavor. Fried tempeh powder contributed richness and oily mouthfeel, which may help improve flavor persistence and perceived body in reduced-sodium matrices. These differences suggest that each tempeh powder type may be suited to different industrial applications. Overripe tempeh powder may be useful for instant noodle seasoning, bouillon, soup bases, and sauces requiring strong umami depth. Roasted tempeh powder may be suitable for gravies, roasted vegetable soups, snack seasonings, and plant-based meat applications. Fried tempeh powder may be more appropriate for richer products such as creamy soups, savory spreads, coatings, and snack formulations.

Roasted tempeh powder (RTP) likely developed flavor through dry-heat reactions, particularly the Maillard reaction [38]. During roasting, free amino acids and reducing sugars react to form a large number of volatile compounds, including pyrazines, pyrroles, furans, aldehydes, and Strecker degradation products. These compounds are commonly associated with roasted, toasted, nutty, cocoa-like, and cooked aromas. Because tempeh already contains increased levels of accessible amino acids after fermentation, it may be an especially reactive substrate for roasting. This likely explains why RTP showed strong roasted aroma and nutty notes in the sensory data. Such cues are highly congruent with savory foods and may enhance perceived richness even without increasing sodium concentration [39].

Fried tempeh powder (FTP) would also undergo Maillard reactions, but under a different thermal environment. Unlike roasting, frying involves direct contact with hot oil, rapid surface dehydration, and simultaneous lipid oxidation or thermal degradation pathways. As a result, FTP may contain not only Maillard-derived roasted compounds, but also lipid-derived aldehydes, ketones, lactones, and other molecules associated with fried, fatty, rich, or crispy aromas [40]. In addition, some oil retention after frying may contribute to mouth-coating sensation and prolonged flavor release. This combination likely explains why FTP was more associated with oily mouthfeel and fuller body than RTP.

The exploratory correlation analysis further supported the sensory interpretation of the reduced-sodium broth formulations. Overall liking showed a strong positive correlation with perceived saltiness intensity (r = 0.83, R^2^ = 0.69, *p* = 0.002) and JAR saltiness proportion (r = 0.88, R^2^ = 0.77, *p* < 0.001), indicating that formulations perceived as sufficiently salty and closer to the ideal saltiness level tended to receive higher consumer acceptance. Overall liking was also positively correlated with umami/savory perception (r = 0.79, R^2^ = 0.62, *p* = 0.004) and meat-like flavor (r = 0.72, R^2^ = 0.52, *p* = 0.013), suggesting that savory sensory attributes may have contributed to liking in the reduced-sodium broth system. In contrast, overall liking was strongly negatively correlated with the weighted mean drop from penalty analysis (r = −0.91, R^2^ = 0.83, *p* < 0.001) and the proportion of “too low” saltiness responses (r = −0.86, R^2^ = 0.74, *p* = 0.001). These results suggest that consumer acceptance was associated not only with sodium level, but also with perceived saltiness adequacy and savory flavor perception. However, because the correlation analysis was exploratory and based on formulation-level means, these relationships should be interpreted as supportive associations rather than evidence of causal sensory mechanisms.

The industrial relevance of tempeh powders is also supported by current consumer interest in fermented, plant-based, and clean-label ingredients [41]. Compared with some conventional sodium-reduction strategies, such as potassium chloride or synthetic flavor enhancers, tempeh powders offer a more food-based approach. They can provide amino acids, peptides, aroma compounds, and plant protein associations in a single ingredient. This may allow manufacturers to position reformulated products not only as “reduced sodium,” but also as containing naturally fermented plant ingredients. Such positioning may be attractive for products targeting health-conscious consumers, especially in markets where tempeh is already familiar.

The findings also highlight that successful sodium reduction depends on maintaining consumer enjoyment. Reduced-sodium products may fail if consumers perceive them as bland and compensate by adding table salt or choosing higher-sodium alternatives. In this study, tempeh powders reduced the penalty associated with “too low” saltiness, indicating that they helped protect liking when sodium was reduced. This is important because products that meet nutritional targets but are not accepted by consumers are unlikely to produce meaningful dietary change.

Nevertheless, commercial translation would require further work. Manufacturers would need to evaluate ingredient stability, batch consistency, cost, allergen labeling because of soybean, and compatibility with existing production processes. The intensity of fermented notes would also need careful optimization, especially for markets less familiar with overripe tempeh. While Indonesian consumers may perceive overripe tempeh as a recognizable savory ingredient, consumers from other cultural backgrounds may respond differently to its fermented aroma. Therefore, cross-cultural validation and dose-optimization studies would be important before wider application.

Overall, this study supports the use of tempeh-derived powders as natural flavor-enhancing ingredients for sodium reduction in savory products. The results suggest that salt reduction does not necessarily have to compromise taste when supported by appropriate fermentation- and processing-derived flavor systems. In particular, overripe and roasted tempeh powders appear to be promising clean-label tools for developing healthier broth, seasoning, sauce, and convenience food products with lower sodium content and maintained consumer acceptance.

## 5. Limitations

Several limitations should be considered when interpreting the findings of this study. First, the experiment was conducted using a clear vegetable broth model. This matrix was intentionally selected because it allows changes in saltiness, umami, aroma, and mouthfeel to be detected more clearly. However, commercial savory products are often more complex and may contain starches, fats, proteins, hydrocolloids, spices, and other flavoring ingredients. These components can influence flavor release, saltiness perception, and overall liking. Therefore, the performance of tempeh powders may differ when applied to products such as instant noodles, sauces, soups, gravies, snacks, or ready meals.

Second, the sensory panel consisted mainly of consumers who were familiar with tempeh and broth/soup products. This is useful for evaluating the Indonesian market context, but it may limit broader generalization. In particular, Indonesian consumers may be more accepting of fermented soybean notes and overripe tempeh character because *tempe semangit* is already known in some local culinary applications. Consumers from other countries, or those with limited exposure to fermented soybean products, may perceive the same fermented aroma as too intense or unfamiliar. Cross-cultural consumer testing would therefore be needed before applying overripe tempeh powder in wider international markets.

Third, this study used only one concentration of tempeh powder, namely 2.0% (*w*/*v*). This level was selected to provide detectable flavor contribution while maintaining acceptable broth appearance and fluidity. However, the optimum dosage may differ depending on product type, salt level, processing method, and target consumer group. Lower concentrations may be sufficient for strongly flavored ingredients such as overripe or roasted tempeh powder, while higher concentrations may be needed for milder fresh tempeh powder. Future studies should evaluate dose–response effects to determine the most effective and acceptable inclusion level.

Fourth, a further sensory limitation is that texture-specific attributes were not fully evaluated. Although oily mouthfeel was included in the RATA terms and viscosity was measured instrumentally, attributes such as body, thickness, mouth-coating, and flavor persistence were not assessed in detail. In addition, the exploratory correlation analysis only provided supportive associations among sensory descriptors and liking, and did not constitute a full preference-mapping or causal analysis of consumer acceptance.

Fifth, although total free amino compounds were measured, the study did not include detailed volatile compound analysis. The sensory results suggest that fermented, roasted, nutty, fried, and meat-like notes contributed to sodium compensation, but the specific aroma-active compounds responsible for these effects were not identified. Further studies using HS-SPME-GC–MS, GC–olfactometry, or aroma recombination approaches would help clarify the chemical basis of the observed sensory differences among fresh, overripe, roasted, and fried tempeh powders.

Finally, this study focused on sensory acceptance and sodium reduction potential, but did not actually evaluate actual dietary behavior. Although the formulations achieved up to 30% sodium reduction in the broth model, it remains unknown whether consumers would maintain acceptance during repeated consumption or compensate by adding salt later. Future studies should therefore include repeated-exposure testing and real-use conditions to better estimate the practical impact of tempeh-based sodium reduction strategies.

## 6. Conclusions

This study demonstrated that tempeh-derived powders can improve the sensory quality of reduced-sodium vegetable broth, with the effect depending on the type of post-fermentation processing applied. Among the tested variants, overripe tempeh powder was the most promising candidate, as it most consistently improved perceived saltiness adequacy, maintained consumer liking, increased the proportion of JAR saltiness responses, and reduced the hedonic penalty associated with insufficient saltiness. However, this effect should be interpreted as sodium compensation rather than direct evidence of a specific saltiness-enhancement mechanism. Such an improved sensory performance may have resulted from the combined contribution of higher free amino compound levels, increased umami intensity, fermented sensory notes, and overall flavor richness. Roasted and fried tempeh powders also provided sensory benefits, and their effects appeared to be associated with different aroma- and mouthfeel-related attributes.

These findings suggest that tempeh-derived powders, particularly overripe tempeh powder, may serve as promising natural ingredients for supporting sodium reduction in savory broth systems. However, the mechanisms underlying these sensory effects were not fully confirmed, as detailed volatile profiling and specific taste-active compound analysis were not conducted. Further studies should evaluate dose–response effects, storage stability, volatile and taste-active compounds, cross-cultural consumer acceptance, and performance in more complex food matrices before broader application.

## Figures and Tables

**Table 1:** Formulation of broth samples.

Sample Code	Broth Base (%)	NaCl (% *w*/*v*)	Salt Level (% of Reference)	Sodium Reduction (%)	Tempeh Powder Type	Tempeh Powder (% *w*/*v*)
CON_100	100	0.80	100	0	–	0
CON_80	100	0.64	80	20	–	0
CON_70	100	0.56	70	30	–	0
TP_80	100	0.64	80	20	Fresh	2
TP_70	100	0.56	70	30	Fresh	2
OTP_80	100	0.64	80	20	Overripe	2
OTP_70	100	0.56	70	30	Overripe	2
RTP_80	100	0.64	80	20	Roasted	2
RTP_70	100	0.56	70	30	Roasted	2
FTP_80	100	0.64	80	20	Fried	2
FTP_70	100	0.56	70	30	Fried	2

**Table 2:** Physical characteristics of broth with tempeh powders.

Sample Code	L*	a*	b*	ΔE*	Viscosity (mPa·s)
CON_100	62.4 ± 0.8 ^a^	1.2 ± 0.1 ^f^	14.8 ± 0.4 ^f^	0	2.05 ± 0.06 ^d^
CON_80	62.7 ± 0.7 ^a^	1.1 ± 0.1 ^f^	14.6 ± 0.5 ^f^	0.5 ± 0.0 ^f^	2.03 ± 0.05 ^d^
CON_70	62.8 ± 0.6 ^a^	1.1 ± 0.1 ^f^	14.5 ± 0.4 ^f^	0.5 ± 0.0 ^f^	2.02 ± 0.05 ^d^
TP_80	58.9 ± 0.8 ^b^	1.7 ± 0.2 ^e^	17.3 ± 0.5 ^e^	4.4 ± 0.1 ^e^	2.42 ± 0.08 ^c^
TP_70	59.1 ± 0.7 ^b^	1.6 ± 0.2 ^e^	17.1 ± 0.5 ^e^	4.2 ± 0.2 ^e^	2.39 ± 0.07 ^c^
OTP_80	57.4 ± 0.8 ^c^	1.9 ± 0.2 ^d^	18.2 ± 0.5 ^d^	6.0 ± 0.2 ^d^	2.56 ± 0.09 ^b^
OTP_70	57.6 ± 0.7 ^c^	1.9 ± 0.2 ^d^	18.0 ± 0.5 ^d^	5.8 ± 0.2 ^d^	2.53 ± 0.08 ^b^
RTP_80	54.6 ± 0.7 ^e^	3.3 ± 0.2 ^a^	22.0 ± 0.6 ^a^	11.1 ± 0.4 ^a^	2.51 ± 0.09 ^b^
RTP_70	54.8 ± 0.8 ^e^	3.2 ± 0.2 ^a^	21.8 ± 0.5 ^a^	10.8 ± 0.3 ^a^	2.48 ± 0.08 ^b^
FTP_80	56.1 ± 0.8 ^d^	2.7 ± 0.2 ^b^	20.1 ± 0.6 ^b^	8.5 ± 0.2 ^b^	2.68 ± 0.10 ^a^
FTP_70	56.3 ± 0.7 ^d^	2.6 ± 0.2 ^b^	19.9 ± 0.5 ^b^	8.2 ± 0.1 ^c^	2.64 ± 0.09 ^a^

Values are presented as mean ± SD (n = 3). Different superscript letters within the same column indicate significant differences among samples based on one-way ANOVA followed by Tukey’s HSD test (*p* < 0.05). ΔE* was calculated relative to CON100 and was not statistically analyzed. Abbreviations: CON = control broth; TP = fresh tempeh powder; OTP = overripe tempeh powder; RTP = roasted tempeh powder; FTP = fried tempeh powder.

**Table 3:** Chemical characteristics of broth with tempeh powders.

Sample Code	pH	Calculated Na Addition (mg/100 mL)	Total Free Amino Compounds (mg GAE/L)
CON_100	6.22 ± 0.03 ^a^	314.7	82.4 ± 3.5 ^e^
CON_80	6.23 ± 0.02 ^a^	251.8	80.8 ± 3.2 ^e^
CON_70	6.22 ± 0.03 ^a^	220.3	79.5 ± 3.7 ^e^
TP_80	6.20 ± 0.03 ^a^	251.8	165.3 ± 5.8 ^d^
TP_70	6.21 ± 0.02 ^a^	220.3	163.8 ± 6.1 ^d^
OTP_80	6.25 ± 0.04 ^a^	251.8	245.6 ± 8.4 ^a^
OTP_70	6.24 ± 0.03 ^a^	220.3	242.2 ± 7.9 ^a^
RTP_80	6.19 ± 0.04 ^a^	251.8	198.8 ± 6.7 ^b^
RTP_70	6.20 ± 0.03 ^a^	220.3	196.5 ± 6.3 ^b^
FTP_80	6.19 ± 0.05 ^a^	251.8	178.4 ± 5.9 ^c^
FTP_70	6.20 ± 0.03 ^a^	220.3	176.9 ± 6.0 ^c^

Values are presented as mean ± SD (n = 3). Different superscript letters within the same column indicate significant differences among samples based on one-way ANOVA followed by Tukey’s HSD test (*p* < 0.05). Abbreviations: CON = control broth; GAE = glutamic acid equivalent; TP = fresh tempeh powder; OTP = overripe tempeh powder; RTP = roasted tempeh powder; FTP = fried tempeh powder.

## Data Availability

The original contributions presented in this study are included in the article. Further inquiries can be directed to the corresponding author.
